# Combined treatment of high‐intensity interval training with neural stem cell generation on contusive model of spinal cord injury in rats

**DOI:** 10.1002/brb3.3043

**Published:** 2023-05-11

**Authors:** Reza Keikhaei, Elahe Abdi, Marzieh Darvishi, Zohreh Ghotbeddin, Hatef Ghasemi Hamidabadi

**Affiliations:** ^1^ School of Medicine Tehran University of Medical Sciences Tehran Iran; ^2^ Isfahan Neurosciences Research Center Isfahan University of Medical Sciences Isfahan Iran; ^3^ Shefa Neuroscience Research Center Khatam Alanbia Hospital Tehran Iran; ^4^ Department of Anatomy, Faculty of Medicine Ilam University of Medical Sciences Ilam Iran; ^5^ Department of Physiology, Faculty of Veterinary Medicine Shahid Chamran University of Ahvaz Ahvaz Iran; ^6^ Stem Cell and Transgenic Technology Research Center Shahid Chamran University of Ahvaz Ahvaz Iran; ^7^ Department of Anatomy & Cell Biology, Faculty of Medicine Mazandaran University of Medical Sciences Sari Iran; ^8^ Immunogenetic Research Center Department of Anatomy & Cell Biology, Faculty of Medicine Mazandaran University of Medical Sciences Sari Iran

**Keywords:** High‐intensity interval training (HIIT), neural stem cells (NSCS), neurospheres

## Abstract

**Introduction:**

Spinal cord injury (SCI) leads to inflammation, axonal degeneration, and gliosis. A combined treatment of exercise and neural stem cells (NSC) has been proposed to improve neural repair. This study evaluated a combined treatment of high‐intensity interval training (HIIT) with NSC generation from adipose‐derived stem cells (ADSCs) on a contusive model of SCI in rats.

**Materials and Methods:**

In vitro, rat ADSCs were isolated from the perinephric regions of Sprague–Dawley rats using enzymatic digestion. The ADSCs were transdifferentiated into neurospheres using B27, EGF, and bFGF. After production of NSC, they were labeled using green fluorescent protein (GFP). For the in vivo study, rats were divided into eight groups: control group, sham operation group, sham operation + HIIT group, sham operation + NSC group, SCI group, SCI + HIIT group, SCI + NSC group, and SCI/HIIT/NSC group. Laminectomy was carried out at the T12 level using the impactor system. HIIT was performed three times per week. To assess behavioral function, the Basso–Beattie–Bresnahan (BBB) locomotor test and H‐reflex was carried out once a week for 12 weeks. We examined glial fibrillary acidic protein (GFAP), S100β, and NF200 expression.

**Results:**

NSC transplantation, HIIT and combined therapy with NSC transplantation, and the HIIT protocol improved locomotor function with decreased maximum H to maximum M reflexes (H/M ratio) and increased the Basso–Beattie–Bresnahan score.

**Conclusion:**

Combined therapy in contused rats using the HIIT protocol and neurosphere‐derived NSC transplantation improves functional and histological outcomes.

## Graphical abstract

Neurosphere‐derived NSC transplantation leads to improved motor recovery and axonal growth in the injured spinal cord through induction of neurotrophic factors and Schwann cell proliferation through exercise along with reducing gliosis.

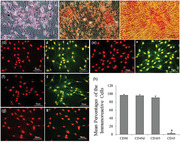

## 1 INTRODUCTION

Spinal cord injury (SCI) is a traumatic neurological condition that is characterized by the complete or incomplete loss of motor, sensory, and autonomic neural functions (Rouanet et al., 2017). SCI is caused by a primary damage mechanism that leads to secondary tissue loss through a cascade of cellular and molecular reactions (Gaudet & Fonken, 2018; Kim et al., 2017). These changes are distinct in two phases: the first is an acute phase that causes edema, hemorrhage, demyelination, inflammation, oxidation, apoptosis, and necrosis of both neurons and oligodendrocytes (Allison & Ditor, 2015; Jendelova, 2018; Kim et al., 2017). The second phase leads to cavity formation, microglial activity, and glial scar formation (astrogliosis). Morphological changes associated with secondary damage are barriers to replacing the cavity with endogenous neural stem cells (NSCs) and axonal regeneration (Akhtar et al., 2008; Darvishi et al., 2014). To improve axonal regeneration and replacement of cell loss following central nervous system (CNS) injury, stem cells, and environmental factors, such as neurotrophins, are critical (Gazdic et al., 2018; Hodgetts & Harvey, 2017; Perea & Araque, 2007). Although promising results have been obtained for the treatment of SCI, there are few to no options to improve functional recovery (Hodgetts & Harvey, 2017; Shahrezaie et al., 2017).

A variety of neurotrophic factors, including nerve growth factor, brain‐derived neurotrophic factor (BDNF), ciliary neurotrophic factor, and neurotrophin 3 (NT3) and 4 (NT4), were capable of inducing axon and dendritic extension (McTigue et al., 1998; Sainath & Gallo, 2015; Tanaka et al., 2000). There was also an effect on neuronal activity, survival, and remodeling. Of these, BDNF improved the regeneration of axonal damage and synaptogenesis. In addition, NT3 and BDNF restrained the formation of a glial scar after CNS injury (Xu et al., 1995; Yan & Wood, 2000), while a combination of NT3/BDNF and basic fibroblast growth factor (FGF2) mediated survival and axon regeneration following optic nerve injury (Blanco et al., 2000). Moreover, cell transplantation has been tested for the replacement of dead cells. Therefore, transdifferentiation of adipose‐derived stem cells (ADSC) was proposed as an appropriate source of neural lineage, since these can be easily obtained and can generate neurotrophic factors, extracellular matrix molecules that promote axonal growth (Mazini et al., 2019; Ohta et al., 2017). One of the limitations of using cell transplantation may be inadequate survival and integration of graft cells. Several studies have described a decrease in the number of graft cells after transplantation in the injury area and then modest functional recovery (Radhakrishnan et al., 2019). Some recent studies have proposed that functional recovery can be promoted in a combination with other therapeutic approaches such as physical exercise. Physical activity can have a positive effect on CNS activity through promotion of synaptic plasticity and survival neurons (Liu et al., 2019; Mattsson et al., 2008; Uysal et al., 2015). Exercise facilitates motor and sensory function as well, and improves the expression of genes and neurotrophic factors such as BDNF and NT3 in the injured spinal cord (Fritsch et al., 2010; Jung et al., 2016). In addition, there is an increased generation of Schwann cells, axonal growth, and suppressed muscle atrophy following CNS injury (Tashiro et al., 2016; Theisen et al., 2017). Schwann cell implantation into a contusion lesion resulted in unregulated expression of neurotrophic factors, myelination, and axon regeneration and promoted motor function (Flora et al., 2013; Golden et al., 2007; Tran et al., 2018). One exercise protocol is high‐intensity interval training (HIIT), which is characterized by high‐intensity exercise associated with short rest intervals. The result of this training strategy is aerobic‐like effects. This exercise protocol is used for different kinds of disorders such as cardiovascular failure, obesity, pulmonary disease, and type 2 diabetes (Engel et al., 2018; Francois & Little, 2015; Ito, 2019). However, the effect of a combined treatment of HIIT protocols with NSC generation from ADSC on the functional recovery of locomotion is not fully understood. Thus, the purpose of this study was to investigate whether HIIT with NSC transplantation would stimulate histological and functional recovery after SCI.

## 2 MATERIALS AND METHODS

### 2.1 Experimental design

Female Sprague–Dawley rats (n = 60; 200 to 250 g) were used in this study. The rats were housed under standard conditions at a temperature of 20 ± 2°C and a light‐dark cycle consisting of 12 h of light and 12 h of darkness. Both in vitro and in vivo studies used the same colony of Sprague–Dawley female rats. This study was approved according to the guidelines of the Ethical Committee at the Ilam University of Medical Science. Four female rats were used for the isolation of ADSC and generation of NSC. A total of 56 female rats were divided randomly into eight groups: control group, sham operation group (laminectomy only or positive control: PC), laminectomy + HIIT (L/HIIT) group, laminectomy + NSC group (L/NSC), SCI group (negative control: NC), SCI + HIIT (SCI/HIIT) group, SCI + NSC (SCI/NSC) group, and SCI/HIIT/NSC group (n = 7 in each group).

### 2.2 Animal model

The rats were anesthetized (80 mg/kg ketamine and 10 mg/kg xylazine, intraperitoneally), and a laminectomy was performed at the T11 level to expose the T12‐L1 spinal cord without the dura matter. A contusion injury was produced on the exposed dorsal surface of the spinal cord. The contusion was carried out by dropping a 10 g rod with a 2.5 mm diameter, from a height of 25 mm. After an injury, muscles and skin were sutured. The rats in the PC group and in the L/NSC and L/HIIT groups received laminectomy without injury.

### 2.3 High‐intensity interval training protocol

One week after surgery, the rats in the exercise groups (L/HIIT, SCI/HIIT, and SCI/HIIT/NSC groups) were trained by swimming as described by Terada et al. (2004). The training session consisted of 14 bouts of 20/s swimming periods with 10/s resting time between each session. This method was performed three times a week on periodical days. Exercise compatibility was assessed at the end of 6 weeks, when all of the rats were investigated in an acute test of swimming while bearing a load of 14% of their body weight.

### 2.4 ADSC isolation

ADSCs were isolated according to the method explained by Darvishi et al. (2020). In brief, adipose tissue was obtained from the abdominal regions of female rats. The specimen was washed with phosphate‐buffered saline (PBS) containing 100 U/mL penicillin and 100 μg streptomycin (Gibco).The adipose tissue was mashed and incubated with collagenase I (Sigma Company: 0.075%) at 37°C for 30–50 min. After digestion of adipose tissue, the suspension was neutralized with 10% FBS in DMEM, then filtered using a 100‐μm mesh and centrifuged at 1200 rpm/min for 10 min. The cell pellets were cultured in a cell culture medium of DMEM for 24 h at 37°C in 5% CO_2_. Nonadherent cells and debris were rinsed, and fresh medium containing 10% FBS was added. When the ADSCs were at 70–80% confluence, they were detached with 0.05% trypsin (Gibco), and cultured on a six‐well culture plate at three to five passages. The ADSC surface markers (CD90 [Abcam,Cambridge, UK; ab225], CD49d [Santa Cruz, sc‐376334 AF594], CD105 [Abcam, ab11414], and CD45 [Abcam, ab10558]) were assessed with immunocytochemistry techniques. In addition, adipogenic differentiation was induced by DMEM containing 10% FBS, 10 nM dexamethasone, 200 mg/mL indomethacin, and 5 mg/mL insulin (Darvishi et al., 2020; Moayeri et al., 2020). After 21 days, the induced adipocytes were stained using oil red stain. Osteogenic induction was performed in DMEM containing 10 nM dexamethasone, 50 mg/mL L‐ascorbic acid, and 10 mM *b*‐glycerophosphate for 3 weeks. Mineralization of the extracellular matrix was displayed using alizarin red. Chondrogenic induction was carried out using 6.25 lg/mL insulin, 6.25 lg/mL transferrin, 1.25 lg/mL bovine serum albumin, 50 lg/mL ascorbic acid, 10–7 M dexamethasone, and 10 ng/mL TGF‐b3 (Sigma–Aldrich, Steinheim, Germany) for 3 weeks, and chondrogenesis was evaluated with 0.1% Safranin O (Darvishi et al., 2020; Moayeri et al., 2020).

### 2.5 Conversion of ADSC into NSC

ADSCs differentiate into NSCs by neurosphere technique. In the first stage, ADSCs of the fourth passage were removed using trypsin and EDTA and plated with serum‐free DMEM/F12 containing 2% B27, 20 ng/mL of the epidermal growth factor (EGF), and 20 ng/mL of the basic fibroblast growth factor (bFGF) (Invitrogen, Paisley, Scotland). After 7 days, the cell aggregates (neurosphere) were harvested into single cells and the dissociated cells were then cultured in a T25 flask (2 × 10^6^ density) in a neurosphere medium with 10% FBS (NSC culture medium). NSCs were cultured on a six‐well plate and immunolabeled with primary antibodies against nestin (Abcam, ab11306), NF 68 (Abcam, ab223343), Neurod (Abcam, ab239955), Sox2 (Abcam, ab92494), Oct4 (Abcam, ab19857), and Neun (Abcam, ab177487) (Darvishi et al., 2020; Moayeri et al., 2020).

### 2.6 Cell labeling and transplantation

Transfection was carried out using the pEGFP‐C1 plasmid reporter gene vector. NSCs were cultured in DMEM without serum, and as the cells reached 80% confluence, transfection was performed with lipofectamine™ 2000 (Qiagen) according to the manufacturer's instructions. At 48 h after transfection, the cells were examined with a fluorescing microscope, and the cells were then subjected to an operation. After 7 days following the contusion, the rats of the NSC groups were reanesthetized and the laminectomy site was reexposed. Green fluorescent protein (GFP)‐labeled NSCs were collected from the cell culture plates and transplanted using a 10 μL Hamilton syringe and a microinjection pump (Stoelting Co.) at a rate of 25 μL/min. The transplants consisted of 1,00,000 cells per 3 μL of PBS at the rostral, epicenter, and caudal regions of the injury site (all injury sites received 3,00,000 cells per 9 μL) (Moayeri et al., 2020).

### 2.7 Basso–Beattie–Bresnahan (BBB) locomotor scale

Functional recovery was evaluated using the Basso–Beattie–Bresnahan (BBB) locomotor scale. The test was carried out 2 days before surgery and again following surgery on days 1, 3, 7, 8, 9, and 14, then once a week for 12 weeks. The BBB test is a standard method to assess hindlimb locomotion in an open field (80 × 130 × 30 cm) with two blind investigators. The BBB test was assessed using the 21‐locomotion scale (0 = flat paralysis and 21 = normal gait) with animal observation for 3 min (Darvishi et al., 2014).

### 2.8 Hoffman reflex

The Hoffman reflex (H‐reflex) was performed as described earlier (Darvishi et al., 2014). Briefly, the ratio of the maximum H to maximum M reflexes (H/M ratio) was done with an electromyography (EMG) or NCV instrument (Cadwell, Series II, USA). The H/M ratio was recorded at preoperation and again at 1, 6, and 12 weeks postoperation. After anesthesia, the sciatic nerve on the left side was exposed 0.5 cm above the nerve bifurcation. To record reflexes, stimulator electrodes (cathode electrode located above the anode) were inserted adjacent to the sciatic nerve. The active, reference, and ground electrodes were located in the plantar muscles, the digital interosseous muscles, and the skin at the base of the tail, respectively. To record the first H‐wave, the sciatic nerve was stimulated with 0.2 ms at 0.1–10 Hz intensity, and then the stimulus decreased the amplitude of the H‐wave until it disappeared. The average H/M ratio was calculated by dividing the maximum value of H‐wave to M‐wave at the maximal stimulus (Darvishi et al., 2014).

### 2.9 Immunofluorescence

#### 2.9.1 Immunocytochemistry

For immunofluorescence, ADSCs and NSCs were seeded on gelatin‐coated coverslips and washed three times with PBS, and then fixed in 4% paraformaldehyde in PBS for 15 min and exposed to 0.1% bovine serum albumin solution containing triton x‐100 (0.3%) for 30 min at room temperature. Next, cells were incubated overnight with primary antibodies that included CD90, CD49, CD105, CD45, Nestin, NF68, Sox2, Oct4, NeuroD, and NeuN (see Table 1). After washing, the cells were incubated with fluorescein isothiocyanate (FITC)‐conjugated secondary antibody at 1:200 dilutions performed at room temperature for 1 h. The samples were then counterstained with propidium iodide (PI) and examined under an inverted fluorescing microscope at 200× magnification (Olympus IX71, Olympus, Tokyo, Japan).

**TABLE 1:** Primary antibodies used in assessment of the adipose‐derived stem cells (ADSC) and the neural stem cells (NSC)

Primary Antibody (P/M)	Titer	Host	Cells	Source (company)	Secondary antibody
**CD105(M)**	1:200	Mouse	ADSC	Millipore	R
**CD49d (P)**	1:300	Rabbit	ADSC	Millipore	G
**CD45 (P)**	Rabbit	1:300	ADSC	Millipore	G
**CD90 (M)**	Mouse	1:300	ADSC	Millipore	R
**Nestin (M)**	Mouse	1:100	NSC	Millipore	R
**NF68 (M)**	Mouse	1:200	NSC	Millipore	R
**Sox2 (P)**	Rabbit	1:200	NSC	Abcam	G
Oct4**(P)**	Rabbit	1:200	NSC	Abcam	G
NeuroD**(M)**	Mouse	1:200	NSC	Millipore	R
NeuN**(M)**	Mouse	1:200	NSC	Millipore	R

The secondary antibodies (Abcam) were rabbit anti‐mouse FITC‐conjugated (R) or goat anti‐rabbit FITC‐conjugated (G); the titers of both secondary antibodies were 1:500, P/M shows monoclonal (M) or polyclonal (P).

#### 2.9.2 Immunohistochemistry

After washing, cross‐tissue sections were exposed to triton x‐100 (0.3%), and blocked in 10% goat serum for 45 min at room temperature. Tissue sections were then placed overnight in primary antibodies including anti‐glial fibrillary acidic protein (GFAP) monoclonal antibody (1:150, Millipore, Germany), anti‐S100β cell monoclonal antibody (1:400, Cosmo Bio Co., Japan) and anti‐neurofilament rabbit polyclonal antibody (1:400, Sigma, USA). The sections were incubated for 2 h with FITC secondary antibodies at 1:400 dilution. The sections were evaluated using a fluorescing microscope, and the intensity was calculated for each image (Image J software 1.43U). The coefficient of variation was then measured (20% for all groups).

### 2.10 Histology of spinal tissue

The animals were fully anesthetized using ketamine (80 mg/kg intraperitoneally; Alfasan Company, Woerden, The Netherlands). Thereafter, rats were perfused with 4% paraformaldehyde and 1.5 cm of the spinal cord at the injury site was dissected and post‐fixed in 10% formalin for 24 h. The sample was then divided into seven segments 2 mm from the center of the injury site. The samples were embedded in paraffin and serial sections were obtained at a 7 μm thickness. The cavity volumes (mm^3^) in 14,000 μm of the spinal cord were evaluated for each specimen. For evaluation of the cavity, volumes in the spinal cord cross‐sections were stained with hematoxylin and eosin. For measuring myelination, sections were stained with Luxol fast blue (LFB). The tissue was deparaffinized and incubated with 0.1% LFB solution at 60°C overnight. The slides were rinsed with a solution of 70% ethanol and distilled water and then differentiated in lithium carbonate solution, dipped in 70% ethyl alcohol for 30 s, and again rinsed with water. The sections were counterstained with 0.1% Cresyl fast violet (10 s) and the slides were dipped in a graded series of alcohols, and twice in 100% xylene. The slides were mounted and observed using a light microscope (Olympus).

### 2.11 Statistical Analysis

For all experiments, the data were analyzed using SPSS 16 (www.spss.com). The ANOVA with Tukey's multiple comparison and repeated measures of the ANOVA followed by the Bonferroni post‐test were used for comparing the groups. The data are expressed as the mean ± standard error of the mean. The significance was accepted for *p* values < .05.

## 3 RESULTS

### 3.1 Rat ADSC characterization

**FIGURE 1:**
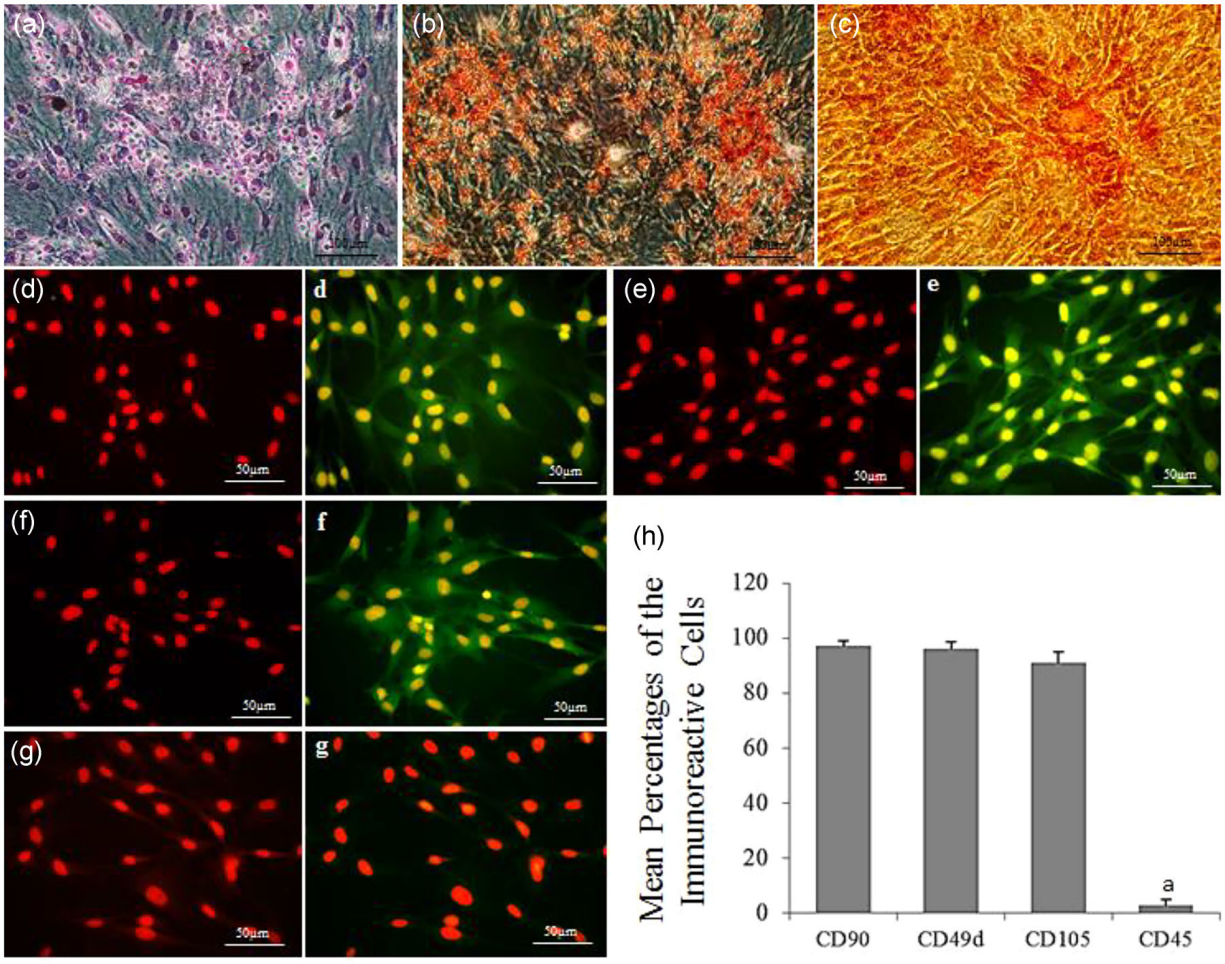
Rat ADSC Characterization. 1a–1c show phase contrast images of the lipogenic, osteogenic, and chondrogenic differentiation of cultured ADSC stained with Oil red, Alizarin red and, safranin O respectively. (d–g) show counterstain (uppercase letters) and immunolabeling figures (lowercase letters) with primary antibodies to CD90, CD49d, CD105, and CD45, respectively. (h) shows a histogram of mean percentages of immunoreactive ADSC to CD90, CD49d, CD105, and CD45. a indicates statistically significant differences from CD105, CD90, and CD49d

Figure 1 shows ADSC differentiation: lipogenic, osteogenic, and chondrogenic (Figure 1a–c). ADSCs were immunoreactive to CD90 (fat‐derived mesenchymal stem cell [MSC] markers), CD49d (fat cell‐specific markers), and CD105 (MSC markers) while they were negatively immunostained to hematopoietic cell markers (CD45). Over 90% of ADSCs were immunoreactive to these markers (Figure 1d–h).

### 3.2 Rat NSC generation and characterization

**FIGURE 2:**
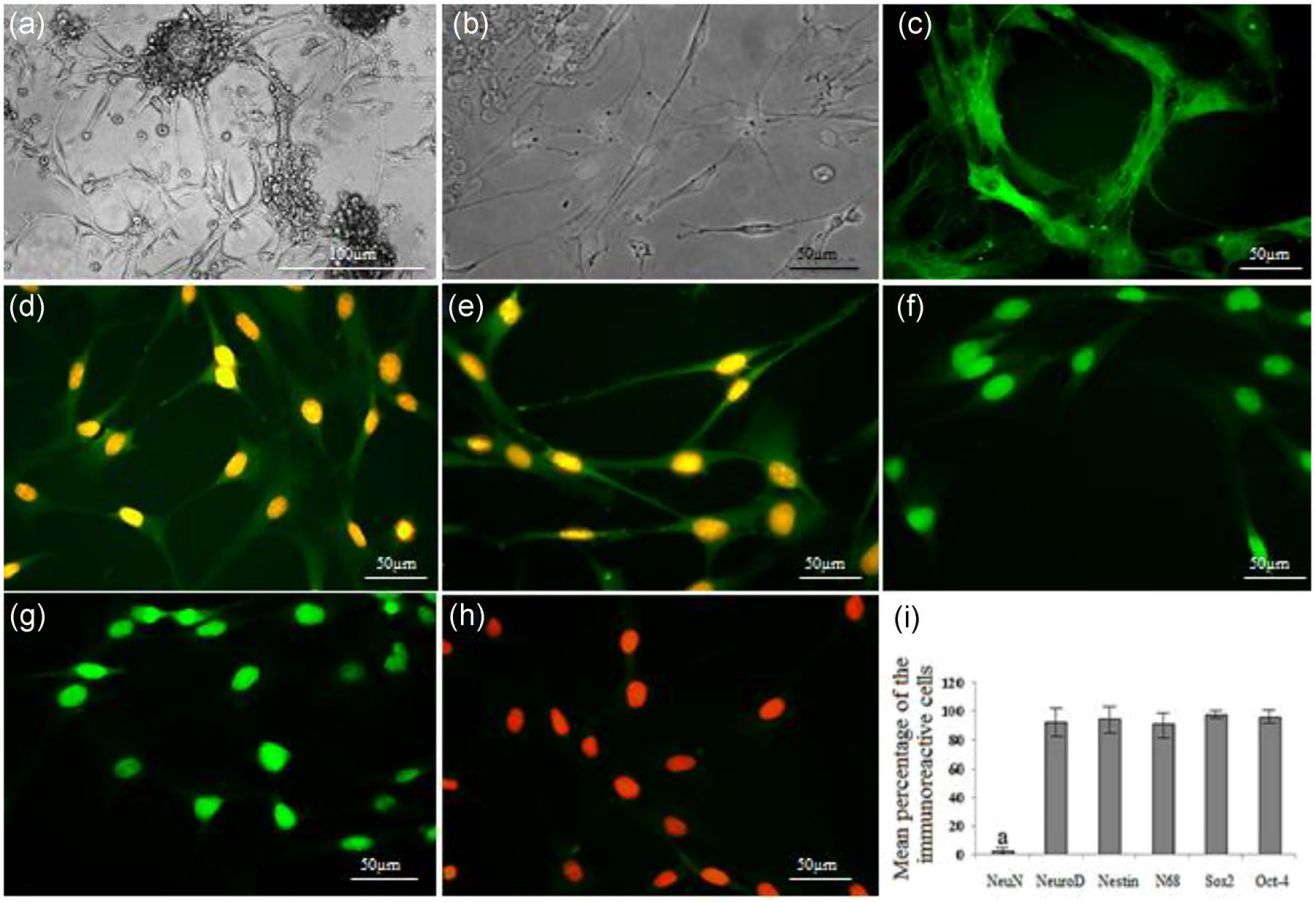
NSC generation and characterization. (a) Phase contrast image of neurospheres generated from the ADSC; (b) morphology of NSC derived from neurospheres. (c–h) Immunolabeling of NSC with primary antibodies (Nestin, NF 68, NeuroD, Sox2, Oct4, and NeuN, respectively). (i) Histogram of percentages of immunoreactive NSC to Nestin, NF 68, NeuroD, Sox2, Oct4, and NeuN. a indicates statistically significant differences NeuN from Nestin, NF 68, NeuroD, Sox2, and Oct4

The morphology of the floating neurosphere is shown in Figure 2a. NSCs were spindle shaped with a long process (Figure 2b). Figure 2c–h illustrates NSC immunostaining using nestin (NSC marker), NF68 (neurofilament 68), NeuroD (undifferentiated neuron marker), Sox2, Oct4 (stemness marker), and NeuN (differentiated neuron markers). The percentages of immunoreactive NSCs to nestin, NF68, Sox2, and Oct4 were significantly higher than the differentiated neuron marker (NeuN), *p* < .05 (Figure 2i).

### 3.3 GFP labeling and transplantation

**FIGURE 3:**
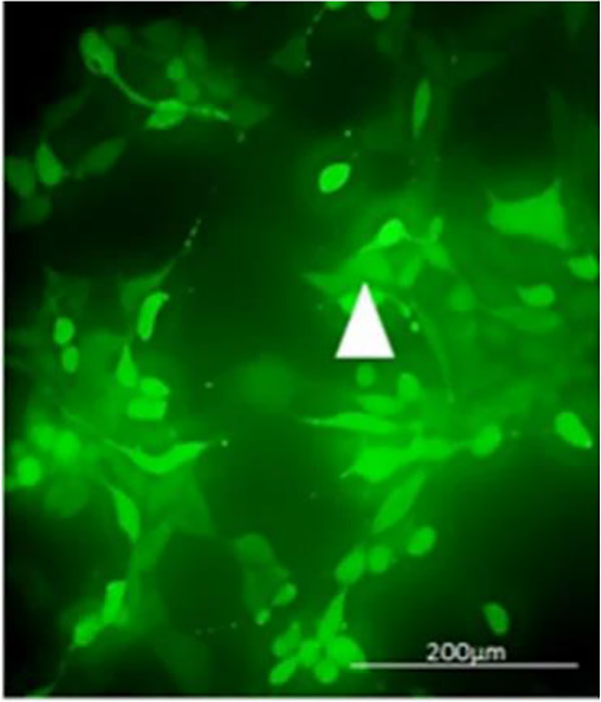
NSCs transfected with pEGFP‐C1 vector (arrowhead); scale bar = 200 μm

Before transplanting cells in vivo, they were labeled with GFP. Figure 3 shows transfection of NSCs using the pEGFP‐C1 plasmid by lipofectamine™ 2000, which shows green fluorescence. The efficiency of nucleofection using the pEGFP‐C1 plasmid was 55.2 ± 1.94%.

**FIGURE 4:**
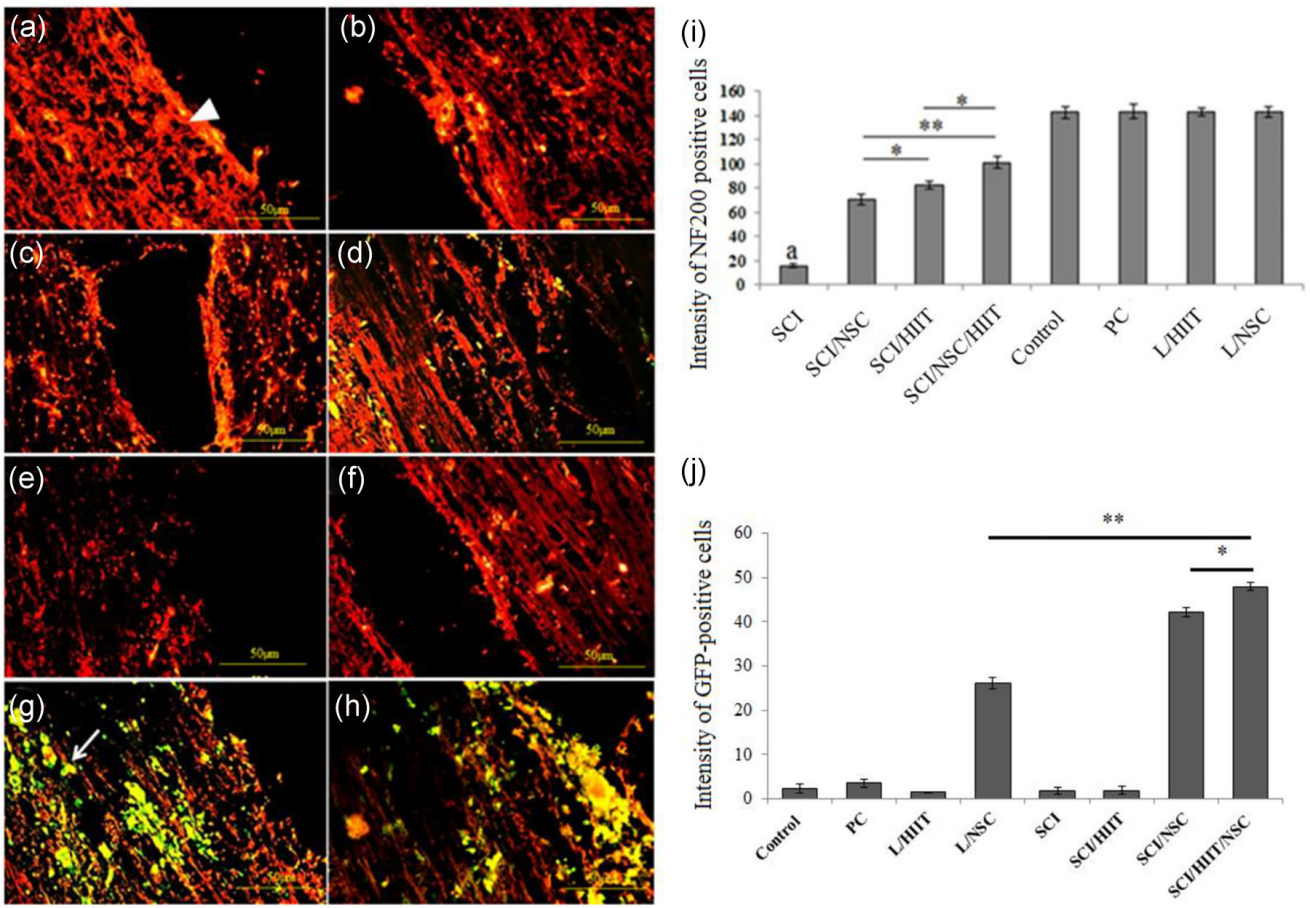
Effects of exercise and NSC transplantation (GFP‐positive cells) on axonal regeneration into the contused spinal cord. (a–h) Expression of NF‐200 in the injured spinal cord following HIIT and NSC transplantation (Normal [control group]), sham operation group [laminectomy or positive control: PC], laminectomy + HIIT [L/HIIT] group, laminectomy + NSC group [L/NSC], SCI group [negative control: NC], SCI + HIIT [SCI/HIIT] group, SCI + NSC [SCI/NSC] group, and SCI/HIIT/NSC group, respectively). White triangle indicates NF200 at the spinal cord (red fluorescence); the arrowheads indicate NSC green fluorescence at the injury site. (i) Relative intensity of NF200 positive cells in the spinal cord. a indicates statistically significant difference from other experimental groups (*p < .05 and **p ≤ .01). (j) Relative intensity of GFP‐positive cells in the spinal cord (*p < .05 and **p ≤ .01)

As shown in Figure 4, GFP‐positive NSCs were detected in the longitudinal section of spinal cord 11 weeks after cell transplantation, demonstrating successful engraftment and survival of the injected NSCs. A high number of GFP‐positive NSCs were integrated after transplantation (see Figure 4d, g, and h). The statistical data for transfecting of NSCs with pEGFP‐C1 are shown in Figure 4j. Immunostaining for NF‐200 was carried out in order to investigate axonal sprouting (Figure 4a‐‐h). The quantitation of immunostaining to NF200 showed that the lowest level was in the SCI groups (*p* < .05), while the highest levels were found in the normal, PC, L/HIIT, and L/NSC groups. The exercise treatment group (SCI/HIIT) showed significantly higher gray level values than the SCI group (*p* < .01) and lower values than the SCI/HIIT/NSC group (*p* < .05). Moreover, the SCI/NSC group showed significantly lower gray level values than the SCI/HIIT and SCI/HIIT /NSC groups (*p* < .01) (see Figure 4i).

### 3.4 BBB locomotor scale

**FIGURE 5:**
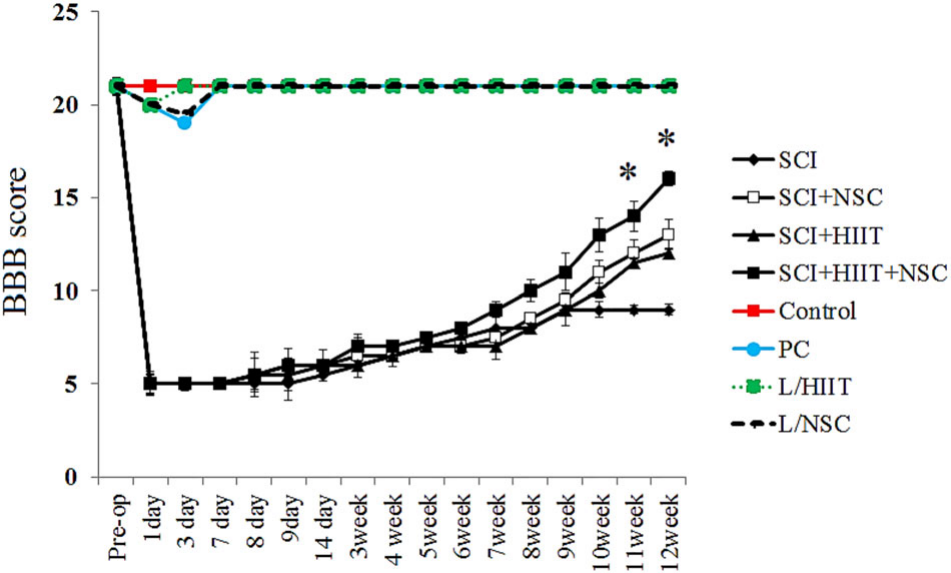
Effect of cell therapy and exercise on locomotor function using BBB score. The data were obtained at days 1, 3, 7, 8, 9 and at weeks 2, 3, 4, 5, 6, 7, 8, 9, 10, 11, and 12. The highest score was 21 (control, PC, L/ HIIT and L/NSC); the lowest was in the contused animals treated with saline (NC). Normal (Control group), sham operation group (PC), laminectomy and HIIT group (L/ HIIT), laminectomy and NSC group (L/NSC), SCI group (negative control: NC), SCI + HIIT group (SCI/HIIT), SCI + NSC group (SCI/NSC), and SCI/HIIT/NSC group. Values are presented as mean ± standard error of the mean. Significance is indicated by *p < .05

The results of the BBB scores are shown in Figure 5 across all eight groups over 12 weeks. The BBB score significantly decreased after SCI, and this condition remained during the experimental study. However, the HIIT, NSC, and combined treatment of HIIT/NSC groups showed increased BBB scores in SCI rats after 12 weeks (*p* < .05). Functional recovery of lower limbs was confirmed by cell therapy and exercise (HIIT). In addition, among three experimental groups (SCI/HIIT, SCI/NSC, and SCI/HIIT/NSC), the highest BBB score was found in the SCI/HIIT/NSC group, which was significantly different from all other experimental groups. The BBB scores for the SCI/HIIT, SCI/NSC, and SCI/HIIT/NSC groups were 12.71 ± 0.24, 13.57 ± 0.14, and 15.28 ± 0.45 at 12 weeks after SCI, respectively.

### 3.5 Hoffman reflex (H‐reflex) analysis

**FIGURE 6:**
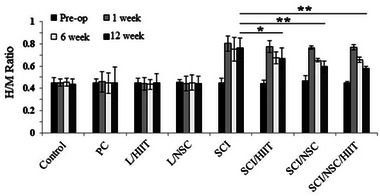
Changes in baseline H/M ratio; pre‐operation (*black*), 1 *(light gray*), 6 (*white*), and 12 (*dark gray*) weeks after injury. There was a significant increase in baseline H/M ratio after 1 week for all SCI groups (SCI+ HIIT, SCI + NSC, and SCI/ HIIT/NSC), but it decreased in the following weeks, and the lowest was seen at 12 weeks post injury (*p < .05 and **p ≤ .01)

The electromyography was done using the H‐reflex as a parameter for evaluating the improvement of the treated animals by an EMG/NCV instrument (Cadwell, Series II). The H/M ratio: H_max_ and M_max_ waves were recorded in order to calculate the H_max_ /H_max_(M_max_), which was done preoperatively, and at 1, 6, and 12 week(s) postoperatively. The effect of exercise and NSC on rat SCI was evaluated by H/M ratio analysis; it was highest at the time of the contusion damage and progressively decreased after HIIT training and NSC implantation. The lowest H/M ratio was 0.579 ± 0.016 in the SCI/HIIT/NSC group, which was significantly different from that seen in the SCI+ HIIT group, and was also significantly lower compared to other times in the same group after injury (Figure 6), but it was not significantly different from the SCI + NSC group.

### 3.6 Gliosis and axon regeneration after SCI

**FIGURE 7:**
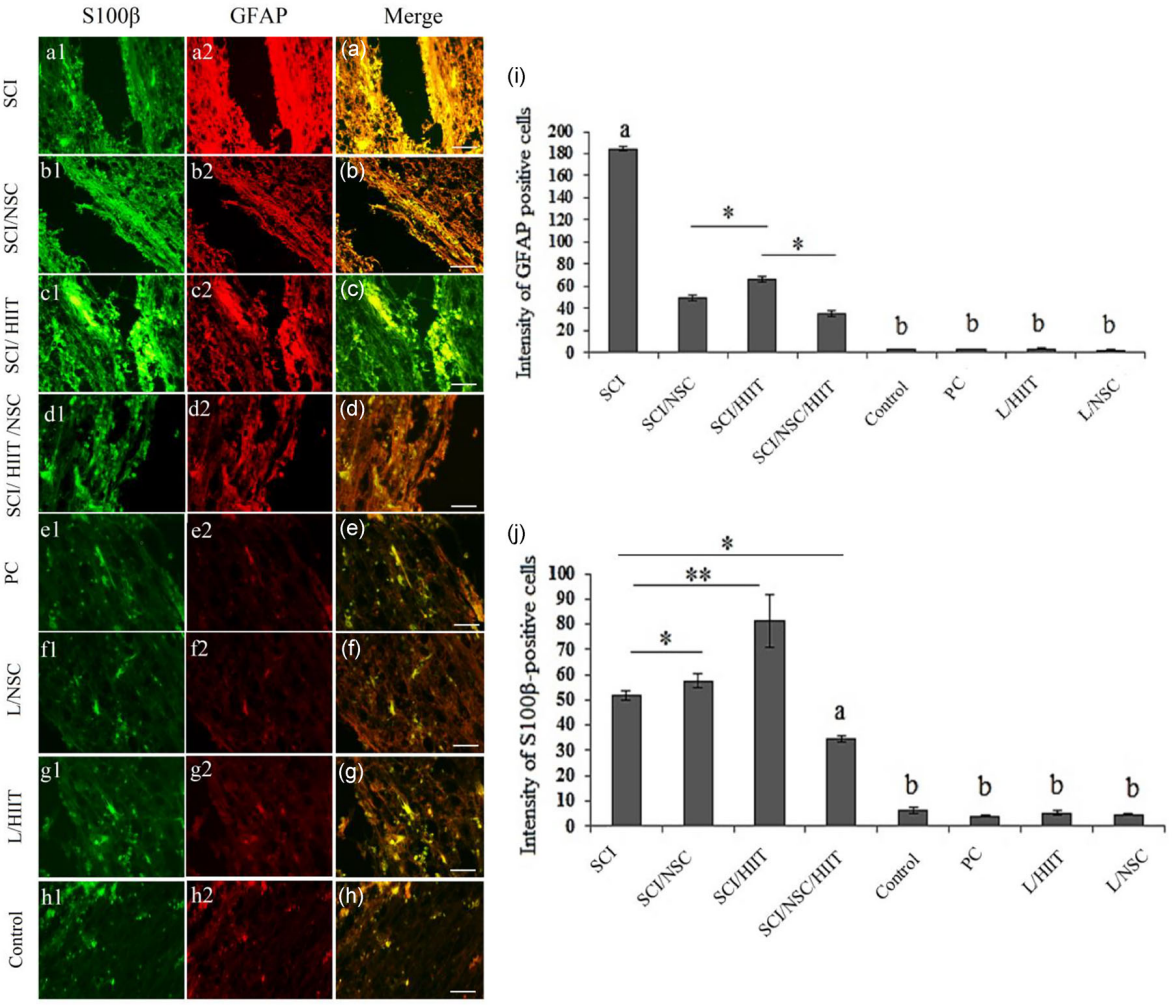
Effects of exercise (HIIT) and NSC transplantation on gliosis (glial fibrillary acidic protein [GFAP]) and Schwann cell proliferation (S100β) into the contused spinal cord. (a1–h1) Tissue sections stained for S100β. (a2–h2) GFAP‐positive cells at the damage site. Alexa red indicate GFAP‐positive cells at the site of the injury. FITC indicate synaptic Schwann cells distributed in the spinal cord. (i) Relative intensity of GFAP‐positive cells in the spinal cord. (j) Relative intensity of S100β‐positive cells in the spinal cord. (*p < .05 and **p ≤ .01). a indicates statistically significant difference compared to the other groups and ***b*** indicates significant difference compared to SCI groups (scale bar 50 μm)

Results also showed the effect of exercise and NSC transplantation on the astrogliosis following SCI by immunostaining for GFAP, a marker for astrocytes. The expression of GFAP‐positive cells declined in both the SCI/HIIT and SCI/NSC groups compared to the untreated group. The significantly lowest level was noticed in the SCI/HIIT/NSC groups (*p* < .05) (Figure 7i).

Figure 7a–h shows Schwann cell migration and integration after exercise and NSC transplantation in SCI via immunostaining for S100β, a marker for Schwann cells. The expression of S100β‐positive cells increased in the SCI groups compared to the normal and sham‐operation groups (Figure 7j; *p* < .001). The relative intensity of S100β within the three treatment groups (SCI/HIIT, SCI/NSC, and SCI/HIIT/NSC) was increased by HIIT in the SCI rats (*p* < .05).

**FIGURE 8:**
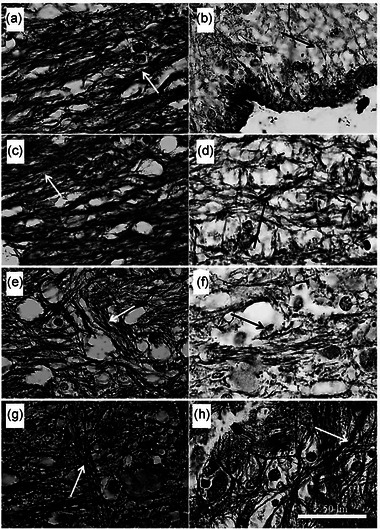
Effect of exercise (HIIT) and NSC transplantation on axonal regeneration in the dorsal columns of the injured spinal cord. Photomicrographs show the longitudinal section from the dorsal columns after silver impregnation staining following HIIT and NSC transplantation. (a, c, e, and g) Normal (control group), sham operation group (PC), sham operation + HIIT group (L/HIIT), and sham operation + NSC group (L/NSC), respectively. (b, d, f, and h) negative control (SCI group: NC), SCI + HIIT group (SCI/HIIT), SCI + NSC group (SCI/NSC), and SCI/HIIT/NSC group, respectively. White arrows indicate neurofibrils packed together, while black arrows show neurofibrils packed loosely (scale bar 50 μm)

The researchers also observed irregular and loose nerve fibers in the untreated group (Figure 8a), while the sham‐operation groups’ fibers were parallel and compact (Figure 8b–e). The exercise‐ and NSC‐treated groups (Figure 8f–h) showed loosely parallel neurofibrils. The combined treatment group (SCI/HIIT/NSC) showed many neurofibrils in the regenerating spinal tissues.

### 3.7 Cavitation

**FIGURE 9:**
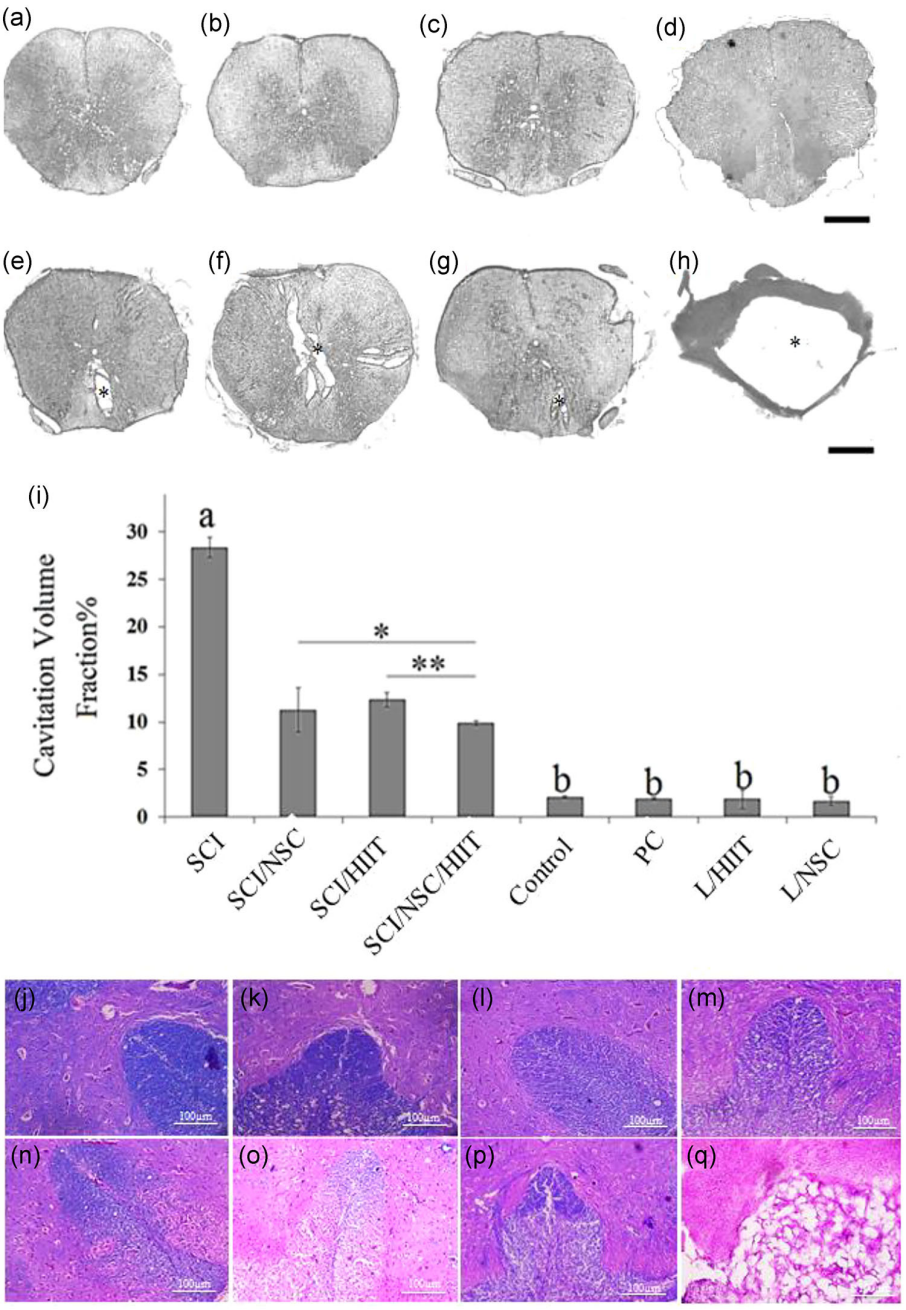
Represents the histological evaluation of cross‐sections from the spinal cord. The cavities in the spinal cord were evaluated after 12 weeks of the spinal cord injury (normal group [a], PC [b], L/HIIT [c], L/NSC [d], SCI/NSC [e], SCI/HIIT [f], SCI/HIIT/NSC [g], and SCI [h]) from the epicenter (asterisk indicates the cavity). Histograms of cavitation volume fraction in spinal cord at epicenter after 12 weeks of injury (i). **a** indicates statistically significant difference from other groups and ***b*** indicates statistically significant differences from SCI, SCI/HIIT, SCI/NSC, and SCI/HIIT/NSC groups (*p < .05 and **p ≤ .01). (j–q) Luxol fast blue stain of posterior column of spinal cord in normal group (j), PC (k), L/HIIT (l), L/NSC (m), SCI/NSC (n), SCI/HIIT (o), SCI/HIIT/NSC (p), and SCI (q), groups (scale bar 100 μm)

At the epicenter of the SCI group, the cavity is large compared with the SCI/HIIT, SCI/NSC, SCI/HIIT/NSC, and sham‐operation groups. Morphometric evaluation shows that the lowest volume density of cavitation was in the SCI/HIIT/NSC group, which was significantly lower than the exercise (SCI/HIIT) and NSC (SCI/NSC) groups. The size of the cavity in the cross‐section of spinal cord was 28.32 ± 1.06% in the SCI group, 11.26 ± 2.35% in the SCI/NSC group, 12.35 ± 0.7% in the SCI/ HIIT group, and 9.1 ± 0.24% in the SCI/HIIT /NSC group (Figure 9a‐I). After SCI, cavity formation was significantly decreased by exercise, cell therapy, and a combination of exercise and NSC graft (*p* < .001). LFB staining in the untreated group showed severe myelin damage in both white and gray matter spatially in the posterior column of white matter (Figure 9j‐q). However, the normal animals, sham operation group (PC), L/HIIT, and L/NSC showed intact myelination (Figure 9j–m), while spinal tissues treated with HIIT and NSC showed remyelination of demyelinated regions (Figure 9n and o). The lowest level of demyelination was seen in the combination therapy group (SCI/ HIIT/NSC) (Figure 9p).

## 4 DISCUSSION

SCI leads to neuronal apoptosis, hemorrhage, and inflammation, cavity formation, loss of trophic factors and glial scarring, resulting in altered neuronal connections and functional disabilities (Gaudet & Fonken, 2018; Kim et al., 2017). Gliosis prevents the growth and regeneration of nerve fibers (Yiu & He, 2006). Stem cell transplantation is a technology used to decrease the inflammatory response, inhibit neural loss, and promote neuronal and axonal regeneration (Zhou et al., 2019). Nevertheless, immunological rejection and poor survival of grafted NSCs are major obstacles to the success of this therapeutic measure (Mothe et al., 2013; Parr et al., 2008). There is little information that explains the cause of transplanted cell death and the effects of environmental factors. Several studies have shown that the death of transplanted NSCs can be induced by reactive nitrogen species or reactive oxygen species (ROS) (Hwang et al., 2014). Exercise can reduce ROS‐related damages through mechanisms involving the antioxidant system, trophic factor expression, and modulation of signaling pathways (Asimakos et al., 2018; Ristow et al., 2009; Simioni et al., 2018). Wang et al. (2006) reported that HIIT enhanced tumor necrosis factor alpha, the transcription factor for BDNF synthesis, and the CREB pathway.

The present study found that a combination of NSCs derived from ADSC and HIIT promoted histological and behavioral recovery in a contusive model of SCI. Therefore, simultaneous cellular and exercise therapy could be a promising method for functional improvement. In recent years, several studies have been conducted on the production of neurospheres from MSC. In 2014, Abbaszadeh and Darabi showed that BMSCs differentiate into the neurosphere and then NSCs using EGF, bFGF, and B27 factors (Abbaszadeh et al., 2014; Mukai et al., 2016). Also, Graf (2011) showed that neurons derived from fibroblasts could be reverted into fibroblasts or even to primitive stem cell populations. For this reason, to stabilize transplanted cells, Monni et al. (2011) and Joo et al. (2012) proposed a neurosphere culture medium. In a previous study, we showed that ADSCs are capable of differentiating into the neurosphere and expressing nestin and stemness markers (Sox2, Oct4, and Nanog). Moreover, NSCs derived from the neurosphere are immunoreactive to nestin (Yang et al., 2015). On the other hand, cell‐nestin positive is known as a lineage‐reprogramming factor with a high differentiation ability and a low risk of tumorigenesis (Bernal & Arranz, 2018; Zhou & Melton, 2008). We carried out an indirect protocol for transdifferentiation as similar as possible to the path of natural differentiation. In the present study, NSCs expressed the embryonic stem cell markers including SOX2, OCT4, and nestin. In addition, no tumorigenesis was detected 12 weeks after transplantation of these NSCs derived from ADSC. Our results showed that GFP‐positive NSCs are able to integrate into the spinal cord, and we confirmed survival and migration. Moreover, transplantation of NSCs led to significantly lower numbers of GFAP immunoreactive cells as well as significantly higher S100β immunodensity of the spinal cord. Riemann et al. (2018) also discussed neural precursor cell transplantation where a significant reduction in astrogliosis and post‐traumatic apoptosis was seen. We observed promotion of functional recovery in NSC‐transplanted animals compare to the untreated group as well as a trend toward a decline in cavity size. Abbaszadeh (2014) reported that neurosphere‐derived oligodendrocyte‐like cells decreased cavity formation at the epicenter of a transplantation area and improved functional recovery in a contusive model of SCI (Abbaszadeh et al., 2014). In this study, we found that neurosphere‐derived NSC transplantation decreased cavity formation and GFAP expression as well as increased S100β followed by axon regeneration compared to the untreated group and this finding is consistent with previous reports (Darvishi et al., 2014; Li & Lepski, 2013). The results of EMG and BBB tests were consistent; our findings showed that the H‐reflex was enhanced after the SCI (Lee et al., 2007). We observed that the H/M ratio increased after SCI and decreased with the implantation of NSCs which is consistent with other studies (Darvishi et al., 2014; Lee et al., 2007). The effects of different types of exercise on functional improvement and expression of neurotrophic factors have been investigated in several models of SCI (Côté et al., 2011; Gómez‐Pinilla et al., 2002). In one study (Heng, 2009), treadmill training increased locomotor function following rat SCI (Heng & de Leon, 2009). In the current study, HIIT decreased the H/M ratio and improved the BBB test 5 weeks after contusion of spinal cord. HIIT promoted the expression of BDNF in the spinal cord and brain (Afzalpour et al., 2015). Moreover, this finding indicates that this neurotrophin, through a tyrosine kinase b receptor (TrkB), increases neurogenesis, axonal regeneration, and synaptogenesis (Tyler & Pozzo‐Miller, 2001; Vaynman et al., 2004). However, BDNF expression decreased in SCI, accompanied with augmentation of axonal sprouting and promotion of this neurotrophin. Ying et al. (2008) reported that the mRNA levels of BDNF and neurotrophin‐3 (NT‐3) were decreased by SCI, and exercise training increased the mRNA levels. Accordingly, it appears that HIIT exercise improves motor recovery after SCI (Leech & Hornby, 2017). These findings are in agreement with the data obtained in this study. Our results showed that SCI led to cavity formation, and HIIT decreases the size of this cavity as well as promoting axonal regeneration and enhancing Schwann cells. Schwann cell proliferation is correlated with both axonal regeneration and migration. These cells increase nerve regeneration by secreting neurotrophic factors. Previous studies have found that implantation of Schwann cells enhanced and guided axonal growth following SCI (Fortun et al., 2009; Imaizumi et al., 2000). In the current study, we observed that HIIT promoted S100β expression while decreasing GFAP expression, which is in agreement with other studies. Neurosphere‐derived NSC transplantation leads to improved motor recovery and axonal growth in the injured spinal cord through the induction of neurotrophic factors and induction of Schwann cell proliferation through exercise, along with reducing gliosis. The simultaneous effects of cell therapy (NSC) and exercise (HIIT) on the survival of neurons and regrowth of axons were greater than either treatment alone.

## AUTHOR CONTRIBUTIONS

The Authors confirm contribution to the paper as follows: study conception, design and data collection: Reza Keikhaei, Elahe Abdi and Marzieh Darvishi. Analysis and interpretation of results: Elahe Abdi and Marzieh Darvishi draft manuscript preparation: Reza Keikhaei, Elahe Abdi, Zohreh Ghotbeddin, Hatef Ghasemi Hamidabadi. All Authors reviewed the results and approved the final version of the manuscript.

## CONFLICT OF INTEREST STATEMENT

Authors declare that they have no conflict of interest.

### PEER REVIEW

The peer review history for this article is available at https://publons.com/publon/10.1002/brb3.3043.

## Data Availability

The data that support the findings of this study are available from the corresponding author upon reasonable request
